# Supplementary material for: Pyruvate kinase directly generates GTP in glycolysis, supporting growth and contributing to guanosine toxicity
Source: mBio. 2025 Feb 25;16(4):e03798-24. doi: 10.1128/mbio.03798-24 (PMC11980595; doi:10.1128/mbio.03798-24)
Supplement: Supplemental Figures — Figures S1–S6. [file mbio.03798-24-s0001.pdf]

```
B. subtilis PK                -MRKTKIVCTIGPASESIEMLTKLMESGMNVARLNFSHGDFEEHGARIKNIREASKKLGK 59
G. stearothermophilus PK      MKRKTKIVCTIGPASESVDKLVQLMEAGMNVARLNFSHGDHEEHGRRIANIREAAKRTGR 60

B. subtilis PK                NVGILLDTKGPEIRTHTMENGGIELETGKELIISMDEVVGTTDKISVTYEGLVHDVEQGS 119
G. stearothermophilus PK      TVAILLDTKGPEIRTHNMENGAIELKEGSKLVISMSEVLGTPEKISVTYPSLIDDVSVGA 120

B. subtilis PK                TILLDDGLIGLEVLDVDAAKREIKTKVLNNGTLKNKKGVNVPGVSVNLPGITEKDARDIV 179
G. stearothermophilus PK      KILLDDGLISLEVNAVDKQAGEIVTTVLNGGVLKNKKGVNVPGVKVNLPGITEKDRADIL 180

B. subtilis PK                FGIEQGVDFIAPSFIRRSTDVLEIRELLEEHNAQDIQIIPKIENQEGVDNIDAILEVSDG 239
G. stearothermophilus PK      FGIRQGIDFIAASFVRRASDVLEIRELLEAHDALHIQIIAKIENEEGVANIDEILEAADG 240

B. subtilis PK                LMVARGDLGVEIPAEEVPLVQKELIKKCNALGKPVITATQMLDSMQRNPRPTRAEASDVA 299
G. stearothermophilus PK      LMVARGDLGVEIPAEEVPLIQKLLIKKCNMLGKPVITATQMLDSMQRNPRPTRAEASDVA 300

B. subtilis PK                NAIFDGTDAIMLSGETAAGSYPVEAVQTMHNIASRSEEALNYKEILSKRRDQVGMTITDA 359
G. stearothermophilus PK      NAIFDGTDAVMLSGETAAGQYPVEAVKTMHQIALRTEQALEHRDILSQRTKESQTTITDA 360

B. subtilis PK                IGQSVAHTAINLNAAAIVTPTESGHTARMIAKYRPQAPIVAVTVNDSISRKLALVSGVFA 419
G. stearothermophilus PK      IGQSVAHTALNLDVAAIVTPTVSGKTPQMVAKYRPKAPIIAVTSNEAVSRRLALVWGVYT 420

B. subtilis PK                ESGQNASSTDEMLEDAVQKSLNSGIVKHGDLIVITAG-TVGESGTTNLMKVHTVGDIVAK 478
G. stearothermophilus PK      KEAPHVNTTDEMLDVAVDAAVRSGLVKHGDLVVITAGVPVGETGSTNLMKVHVISDLLAK 480

B. subtilis PK                GQGIGRKSAYGPVVVAQNAKEAEQKMTDGAVLVTKSTDRDMIASLEKASALITEEGGLTS 538
G. stearothermophilus PK      GQGIGRKSAFGKAVVAKTAEEARQKMVDGGILVTVSTDADMMPAIEKAAAIITEEGGLTS 540

B. subtilis PK                HAAVVGLSLGIPVIVGLENATSILTDGQDITVDASRGAVYQGRASVL 585
G. stearothermophilus PK      HAAVVGLSLGIPVIVGVENATTLFKDGQEITVDGGFGAVYRGHASVL 587
```

FIG S1 Alignment of *B. subtilis* pyruvate kinase and *G. stearothermophilus* pyruvate kinase sequences. Suppressor mutations are labeled magenta.

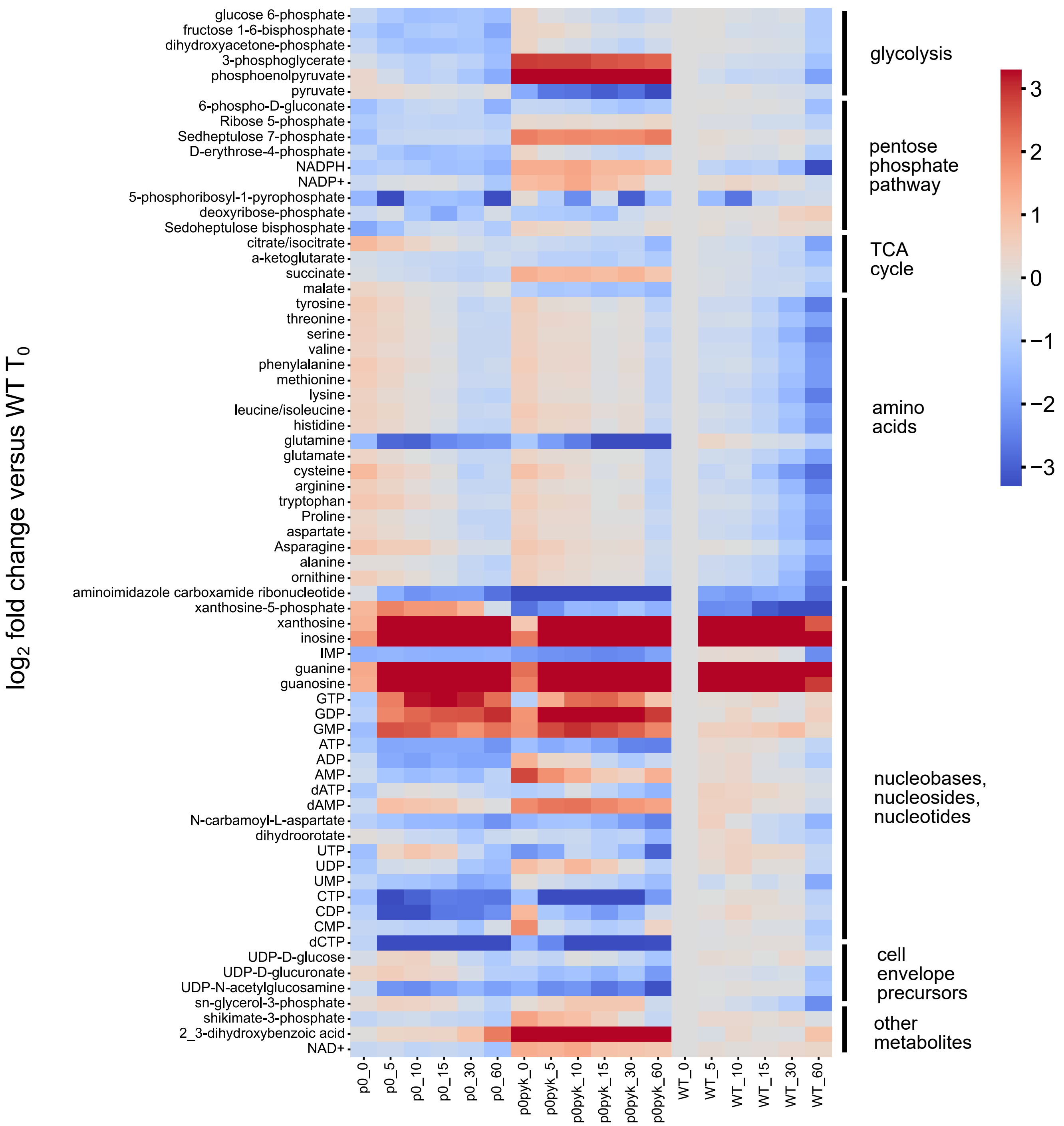

FIG S2 Heatmap of metabolite levels after guanosine treatment in wild type, (p)ppGpp^0 and (p)ppGpp^0 Δ*pyk* strains. The color shows the log2 fold change relative to the metabolite levels in WT before guanosine treatment (WT_0).

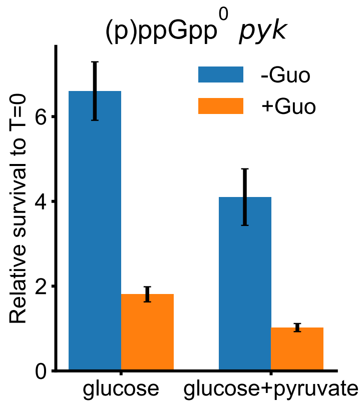

FIG S3 Addition of pyruvate does not abolish the suppression of guanosine toxicity in (p)ppGpp^0 strain. Relative survival of (p)ppGpp^0 *pyk*^{G318E} after guanosine treatment in media supplemented glucose (normal S7+CAS media) or glucose and pyruvate.

nucleotide precursors

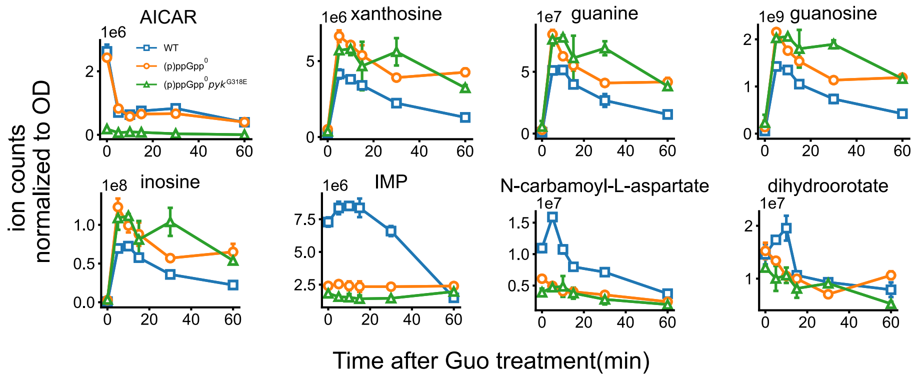

FIG S4 Nucleotide precursors level changes after guanosine treatment in wild type, (p)ppGpp^0 and (p)ppGpp^0 *pyk^{G318E}* over time. Guo: guanosine.

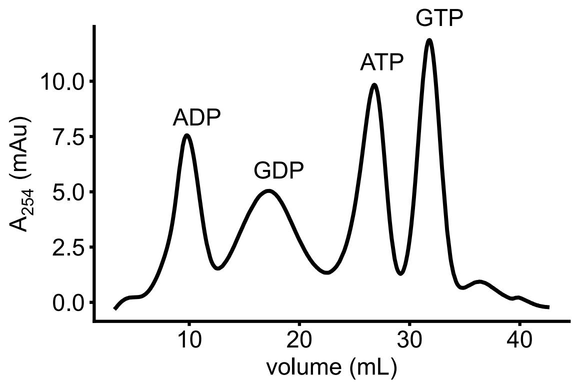

FIG S5 Separation and quantification of nucleotide products in pyruvate kinase reactions. ADP, GDP, ATP and GTP standards can be separated with ion exchange chromatography.

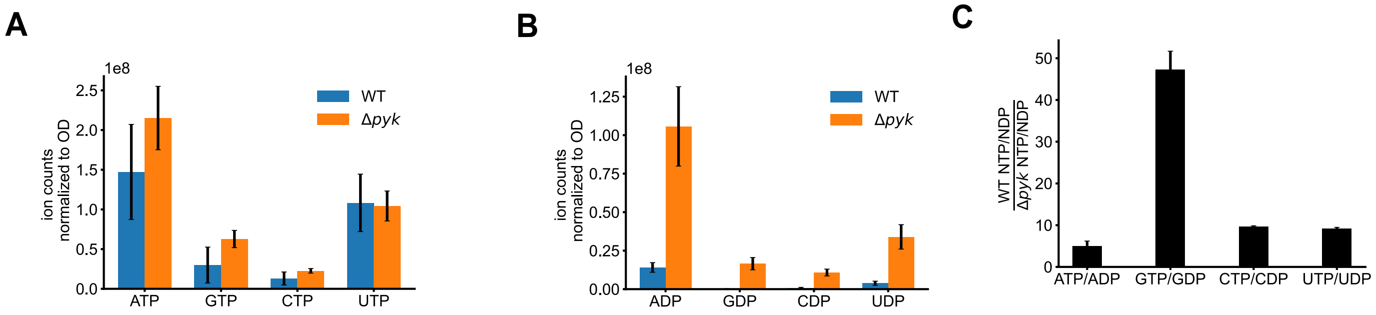

FIG S6 Deletion of *pyk* leads to accumulation of different NDPs to different extents. NTP levels (A) and NDP levels (B) in WT and Δ*pyk* measured by LC-MS. (C) The ratio of NTP/NDP between WT and Δ*pyk*.
